# Supplementary material for: Vitamin D Modified DSS-Induced Colitis in Mice via STING Signaling Pathway
Source: Biology (Basel). 2025 Jun 18;14(6):715. doi: 10.3390/biology14060715 (PMC12190092; doi:10.3390/biology14060715)
Supplement: Supplementary file 1 [file biology-14-00715-s001.zip › Supplementary Materials S1: 16s-report of gut microbiota/Groups/heatmap/Group/non_cluster_norm_100log10.pdf]

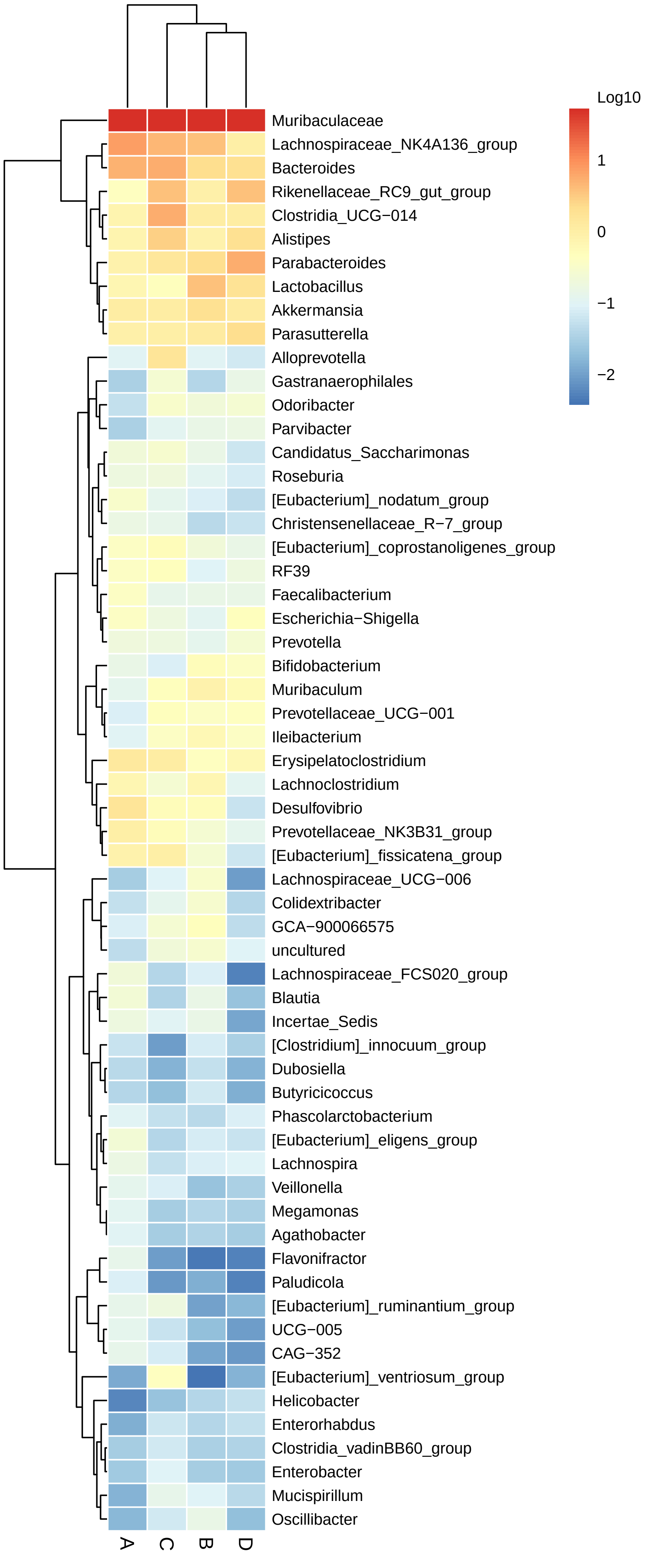

Log10
1
0
−1
−2
Muribaculaceae
Lachnospiraceae_NK4A136_group
Bacteroides
Rikenellaceae_RC9_gut_group
Clostridia_UCG−014
Alistipes
Parabacteroides
Lactobacillus
Akkermansia
Parasutterella
Alloprevotella
Gastranaerophilales
Odoribacter
Parvibacter
Candidatus_Saccharimonas
Roseburia
[Eubacterium]_nodatum_group
Christensenellaceae_R−7_group
[Eubacterium]_coprostanoligenes_group
RF39
Faecalibacterium
Escherichia−Shigella
Prevotella
Bifidobacterium
Muribaculum
Prevotellaceae_UCG−001
Ileibacterium
Erysipelatoclostridium
Lachnoclostridium
Desulfovibrio
Prevotellaceae_NK3B31_group
[Eubacterium]_fissicatena_group
Lachnospiraceae_UCG−006
Colidextribacter
GCA−900066575
uncultured
Lachnospiraceae_FCS020_group
Blautia
Incertae_Sedis
[Clostridium]_innocuum_group
Dubosiella
Butyricicoccus
Phascolarctobacterium
[Eubacterium]_eligens_group
Lachnospira
Veillonella
Megamonas
Agathobacter
Flavonifractor
Paludicola
[Eubacterium]_ruminantium_group
UCG−005
CAG−352
[Eubacterium]_ventriosum_group
Helicobacter
Enterorhabdus
Clostridia_vadinBB60_group
Enterobacter
Mucispirillum
Oscillibacter
A
C
B
D
